# Dyspnea is related to clinical outcomes in patients weaning from invasive mechanical ventilation with tracheostomy: a multicenter prospective study

**DOI:** 10.1186/s13054-025-05734-8

**Published:** 2026-01-08

**Authors:** M. L. Janssen, H. Endeman, Z. Yang, J. H. Elderman, M. Goeijenbier, T. Dongelmans, H. Moeniralam, J. Rozendaal, A. J. A. M. van Hees, J. D. Workum, E. A. N. Oostdijk, P. Petersen, D. van Nieuwenhuizen, T. van Zuylen, A. De Bie Dekker, I. H. F. Herold, S. Stads, S. Achterberg, A. Osinski, L. Heunks, E-J. Wils

**Affiliations:** 1 Intensive Care, Franciscus Gasthuis & Vlietland, Kleiweg 500, 3045 PM, Rotterdam, The Netherlands; 2 Intensive Care, Erasmus MC, Rotterdam, The Netherlands; 3 Pulmonary Medicine, Erasmus MC, Rotterdam, The Netherlands; 4 Intensive Care, OLVG, Amsterdam, The Netherlands; 5 Department of Epidemiology and Biostatistics, Erasmus University Medical Center, Rotterdam, The Netherlands; 6 Intensive Care, IJsselland Ziekenhuis, Capelle a/d IJssel, The Netherlands; 7 Intensive Care, Spaarne Gasthuis, Haarlem, The Netherlands; 8 Intensive Care, Sint Antonius Ziekenhuis, Nieuwegein, The Netherlands; 9 Intensive Care, Elizabeth-Tweesteden Ziekenhuis, Tilburg, The Netherlands; 10 Intensive Care, Rijnstate Ziekenhuis, Arnhem, The Netherlands; 11 Intensive Care, Jeroen Bosch Ziekenhuis’s, Hertogenbosch, The Netherlands; 12 Intensive Care, Catharina Ziekenhuis, Eindhoven, The Netherlands; 13 Intensive Care, Anna Ziekenhuis, Geldrop, The Netherlands; 14 Intensive Care, Ikazia Ziekenhuis, Rotterdam, The Netherlands; 15 Intensive Care, HMC Westeinde, Den Haag, The Netherlands; 16 Intensive Care, Maxima Medisch Centrum, Veldhoven, The Netherlands; 17 Intensive Care, Radboud UMC, Nijmegen, The Netherlands

**Keywords:** Weaning, Dyspnea, Tracheostomy, Weaning failure, Post-ICU sequalae

## Abstract

**Background:**

Tracheostomized critically ill patients weaning from invasive mechanical ventilation (IMV) are at risk for dyspnea. This study aimed to assess the prevalence and severity of dyspnea during tracheostomized weaning, its impact on weaning outcomes, and its association with psychological outcome and health-related quality of life (HR-QoL) after Intensive Care unit (ICU)-discharge.

**Methods:**

A prospective observational study in tracheostomized patients weaning from mechanical ventilation was performed in 13 hospitals in the Netherlands. Main exclusion criteria were tracheostomy for airway obstruction and neuromuscular disease. Dyspnea was assessed daily during mechanical ventilation and weaning. The primary endpoint was the number of weaning days with dyspnea. Main secondary endpoints were dyspnea severity measured using a visual analog scale (D-VAS), weaning success, post-traumatic stress disorder (PTSD) related symptoms and HR-QoL evaluated using IES-R and EQ-5D questionnaires 90 days post- ICU, respectively.

**Results:**

From April 2023 to June 2024, 156 patients were included; 130 (83%) were successfully weaned. The median weaning duration was 10 [8−15] days, with a median of 3 [2−6] days with dyspnea per patient. Dyspnea affected 58% of patients, with a median D-VAS score of 6 [5–7]. Dyspnea was associated with longer weaning duration and reduced weaning success (hazard ratio 0.37, *P* < 0.001). The number of days with dyspnea correlated significantly with IES-R (linear regression coefficient (β) 2.42; *P* = 0.02) and EQ-5D utility score (-0.025; *P* = 0.03).

**Conclusion:**

Dyspnea in tracheostomized critically ill patients is common during weaning and associated with prolonged weaning, reduced weaning success, increased PTSD-symptoms and decreased HR-QoL after ICU discharge.

**Supplementary Information:**

The online version contains supplementary material available at 10.1186/s13054-025-05734-8.

## Introduction

Weaning from invasive mechanical ventilation (IMV) is a pivotal and challenging phase during critical illness, often marked by significant patient burden and resource use [1–5]. Intubated patients are at risk for dyspnea [6]. Prolonged weaning from IMV frequently involves tracheostomy placement to facilitate weaning and patient comfort [1, 3, 7]. However, tracheostomized patients may be even more vulnerable to distressing experiences like dyspnea due to their prolonged and conscious exposure during weaning [8–11].

Despite the substantial influence on patients and ICU resources, studies on dyspnea during the tracheostomized weaning phase remain limited [7, 12]. Moreover, patient-centered outcomes and post-ICU sequelae are increasingly acknowledged as relevant outcomes for critically ill patients [10, 13–17]. Currently, the prevalence and severity of dyspnea in tracheostomized patients during weaning are unknown and the impact of dyspnea on patient-centered and clinical weaning outcomes remain incompletely understood. We hypothesized that tracheostomized patients are at particular risk to experience dyspnea.

The primary objective was to determine the burden of dyspnea during tracheostomized weaning, and the secondary objective was to evaluate the association between dyspnea and weaning outcomes.

## Methods

### Study design and setting

This prospective observational multicenter study was performed in the ICUs of 13 hospitals in the Netherlands. In all participating centers the approach to tracheostomized weaning involved disconnection sessions without ventilatory support, gradually increased in duration until patients were able to breathe independently [18]. Daily weaning management, including the decision to start weaning, the applied respiratory support regimen between and during disconnection and the initiation and termination of disconnection sessions were left to the discretion of the treating clinical team. In the Dutch healthcare setting, discharge to long-term ventilation units rarely occurs in patients without neuromuscular disease. Further information on the local facilities and weaning practice is detailed in the online supplements Table S1 and S2.

The study was performed in accordance with the ethical principles for medical research written in the Declaration of Helsinki. Written informed consent was obtained from all participants or their legal representatives. This study was approved by the local Medical Ethics Committee (MEC-U number W23.001) and prospectively registered (clinicaltrials.gov identifier NCT05906888, Registration date: 5 October 2023). This report adheres to the Strengthening the Reporting of Observational Studies in Epidemiology guidelines (checklist in the supplementary material) [19].

## Participants

The study inclusion criteria were adult patients weaning from IMV with tracheostomy. The indication to perform a tracheostomy was at the discretion of the treating team of clinicians. The study exclusion criteria were: (1) Tracheostomy primarily indicated for upper airway obstruction or to secure airway patency due to persistent stupor/coma, (2) Neuromuscular disease in medical history or diagnosed during current admission (excluding ICU-acquired weakness); (3) Using extracorporeal life support (ECMO or ECCO_2_R) during tracheostomized weaning; (4) Receiving chronic positive pressure respiratory support at home (excluding night-time continuous positive airway pressure for obstructive sleep apnea) (5) Impaired verbal communication prior to admission due to deafness, blindness, or inability to understand the Dutch or English language. Eligible patients were included on the day that tracheostomy was performed.

### Outcomes and endpoints

The primary endpoint was the number of weaning days with reported dyspnea within the first 28 days of the tracheostomy weaning phase. Secondary endpoints were the daily dyspnea severity score, weaning duration, weaning success, and post-ICU survival and psychological symptoms at 90 days after ICU discharge. Tertiary endpoints were the duration of the period with tracheostomy in situ, ICU-, in-hospital, and 28 days survival rate, and the number of endotracheal suctioning maneuvers performed during the weaning phase.

The tracheostomized weaning phase was defined as from the first disconnection after tracheostomy placement until liberation from IMV. Patients were considered liberated from IMV when they were disconnected from IMV for 7 consecutive days or discharged alive from ICU without IMV requirement, whichever came first [20, 21]. Weaning success was defined as discharged alive from ICU without need for IMV, with or without tracheostomy. Post-ICU psychological symptoms (anxiety, depression, and PTSD) and health-related quality of life (HR-QoL) were assessed using validated questionnaires. PTSD symptoms were assessed using the Impact of Event Scale-Revised (IES-R) [22, 23] and probable PTSD was defined as a IES-R total score ≥ 22. Anxiety and depression symptoms were evaluated using the Hospital Anxiety and Depression Scale (HADS) [24, 25]; Probable anxiety and depressive disorder were defined by a score of ≥ 8 on the anxiety or depression items. HR-QoL was assessed using the European Quality of Life 5D (EQ-5D) and the European Quality of Life VAS (EQ-VAS) questionnaires [26, 27].

### Study procedures

The daily dyspnea data collection window and methods are described shortly below, and in detail in the supplementary material (Figure S1). Dyspnea was evaluated daily within the first 28 days of the tracheostomized weaning phase using methods described by others (Figure S2) [6, 13, 28]. In short, first the ability of a patient to communicate was assessed based on a negative delirium screening (negative Confusion Assessment Method for the Intensive Care Unit (CAM-ICU) or Intensive Care Delirium Screening Checklist (ICDSC) score < 4) [29, 30]) and ruling out sedated or agitated mental state (Richmond Agitation and Sedation Scale (RASS) score between − 2 and + 2 [31]). Communicative patients were asked for the presence and severity of dyspnea while on IMV and at the end of disconnection session. The presence of dyspnea was assessed using a binary question (‘Do you experience shortness of breath or trouble breathing at this moment?’). Presence of dyspnea was defined as an affirmative answer to this question; consequently, a day with dyspnea was defined as presence of dyspnea prior to and/or at the end of disconnection. Then, patients were asked to rate their dyspnea severity on a D-VAS, ranging from 0 (no dyspnea) to 10 (worst imaginable dyspnea (Figure S3).

Patients were followed up for 90 days after ICU discharge to determine the total duration of IMV and tracheostomy in situ, hospital- and ICU admission duration. At 90 days after ICU discharge the questionnaires to evaluate symptoms were obtained.

### Sample size and statistical analysis

Prior to this study, data on the primary endpoints in patients weaning from IMV with tracheostomy were unavailable to perform a formal sample size calculation upfront. This observational study therefore used a convenience sample of 150 patients. Statistical methods are detailed in the supplementary material. Analyses were performed using R version 4.4.1.

Dyspnea was reported via three different expression modes:

- Dyspnea prevalence: The proportion of communicative patients who reported dyspnea at any time during the tracheostomized weaning phase.
- Cumulative incidence of dyspnea: the number of communicative weaning days with dyspnea, as scored prior to and/or during the disconnection session.
- Dyspnea density: the rate of days able to communicate with dyspnea, calculated as the cumulative number of days with dyspnea divided by the total communicative weaning days.

Continuous variables were presented as means with standard deviation (SD) or medians with quartiles based on normality of distribution, which was assessed with Shapiro-Wilk tests. Categorical variables were expressed as frequencies and percentages. Between-group comparisons were evaluated with χ2 or Fisher exact test (categorical variables) or the student t-test or Mann-Whitney U test, as appropriate (continuous variables). A p-value less than 0.05 (two-tailed) was considered as statistically significant.

The association between dyspnea in the first 28 day of the tracheostomy-facilitated weaning phase, weaning duration and weaning success was analyzed with joint models, controlling for missing data at random, such as due to non-communicative state [32]. The joint models controlled for the competing risk of death and weaning success. The survival component of the joint models was a Cox proportional hazard model evaluating the weaning time and success. The longitudinal component of the joint models was a generalized linear mixed model with repeated assessments of dyspnea prior to and/or during the disconnection as dependent outcome and time as independent variables, with random intercept and slope. The output of each joint model was a single coefficient, which was exponentiated into a Hazard ratio, representing the association between repeated instances of dyspnea and both the weaning duration and weaning outcome.

To evaluate associations between dyspnea during weaning and psychological and HR-QoL outcomes regression analyses were used. Linear regression was applied for continuous outcomes and logistic regression was used for binomial outcomes. The models assessed the relationship between the dependent variables and the number of days with dyspnea, controlling for weaning duration by including the number of communicative weaning days as an independent variable.

## Results

Between April 2023 and June 2024, 156 patients were included in 13 centers. Baseline characteristics and clinical outcomes are depicted in Table 1 and Table S3. One-hundred thirty (83%) patients were successfully weaned from IMV, of which 25 (16%) were discharged from ICU with tracheostomy in situ. Twenty-six (17%) patients died. The median duration of the tracheostomy-facilitated weaning was 10 [8; 15] days; 9 [8; 15] days in patients who were successfully liberated from IMV, and 13 [9; 20] days in patients who died.

## Prevalence and severity of dyspnea

**Fig. 1:**
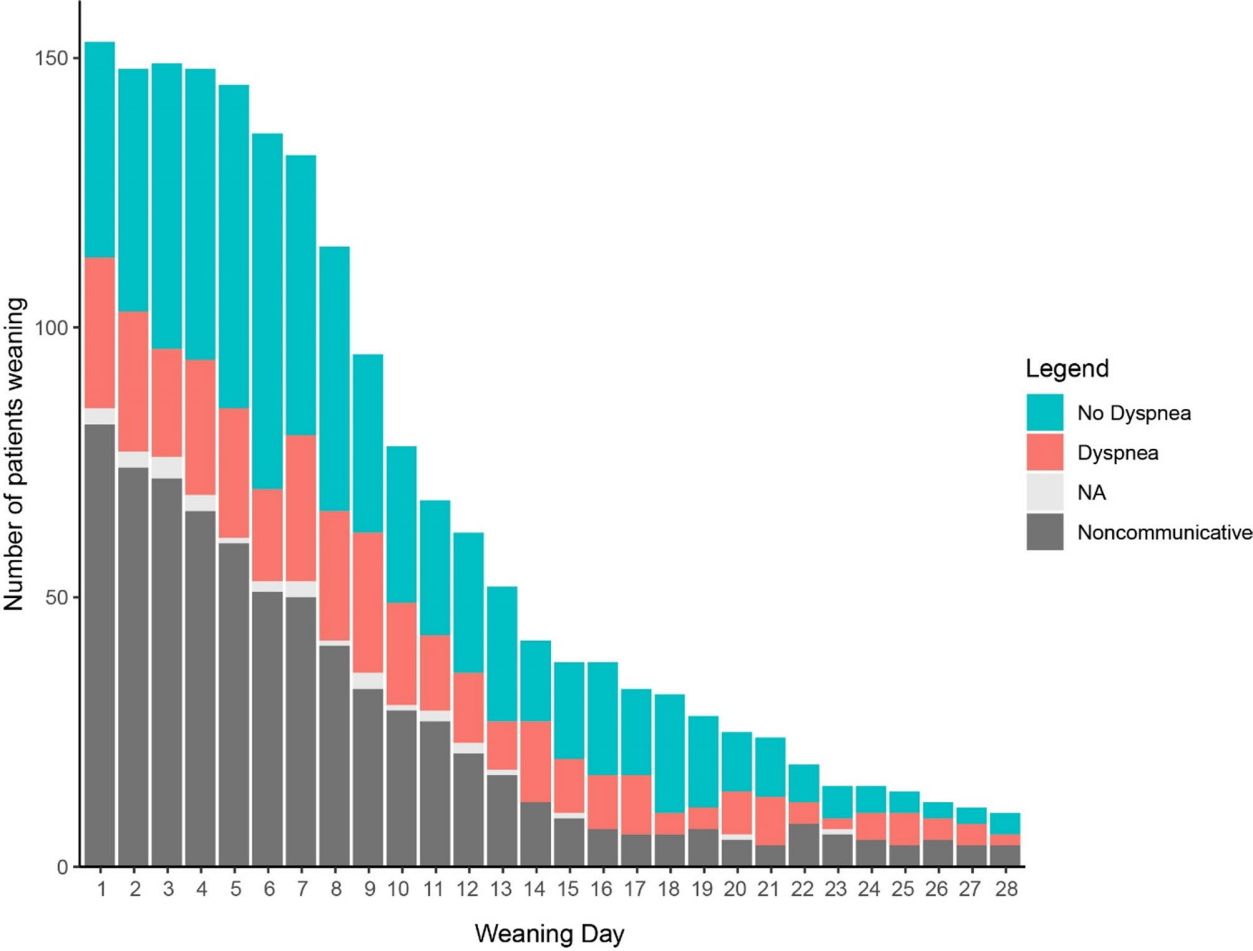
Dyspnea presence during first 28 days of tracheostomized weaning. The results of dyspnea assessments per weaning day over time in patients who received invasive mechanical ventilation in the 24 h prior to dyspnea assessment (i.e. weaning patients). NA: not available

Of the 156 included patients, 5 (3%) were unable to communicate throughout the 28-day observational period. Among the remaining 151 patients able to communicate at least on one day during weaning, 1837 dyspnea assessments were performed in total. In 715 instances (39%) dyspnea assessment could not be performed due to a non-communicative state (Fig. 1). Among the 1122 assessments completed in communicative patients, dyspnea was reported in 370 (33%) cases either on IMV or at the end of the disconnection session (Table 2). Patients used opioids on the day of dyspnea assessments in 57%. Dyspnea was more frequently reported at the end of the disconnection than on IMV prior to the disconnection session (26% vs. 15%, *p* < 0.001). Data on dyspnea presence were missing in 32 (3%) and the D-VAS was missing in 379 (34%) out of 1122 assessments in communicative patients (Table 2).

Subsequently, a repeated measure approach was applied to the dyspnea assessments to account for within-subject correlation and unequal number of dyspnea assessments per patient. Dyspnea prevalence was 58%, with 88 out of 151 communicative patients reporting dyspnea on at least one day during IMV and/or at the end of disconnection sessions. The median number of communicative weaning days per patient was 7 [4; 12], with dyspnea reported on a median of 3 [2; 6] days (Table 3). The median cumulative dyspnea density was 0% [0; 29] during IMV, 17% [0; 40] at the end of disconnection sessions and 22% [0; 50] when considering both during IMV and/or the end of disconnection sessions. The D-VAS was 6 [5; 7] in patients who reported dyspnea during the assessment and 0 [0; 3] in patients who did not report dyspnea (Figure S4). A D-VAS above 3.0 was more frequently present at the end of disconnection than during IMV (28% vs. 20%, *p* < 0.001).

To determine differences between patients with or without significant dyspnea burden, patients were compared based on median dyspnea density. Only the level of support on IMV at the first disconnection session was higher in patients with high dyspnea density: pressure support level: 10 [8; 12] vs. 8 [6; 10] cm H_2_O (*P* = 0.01, Table S4).

### Association between dyspnea and 28-day weaning outcome

**Fig. 2:**
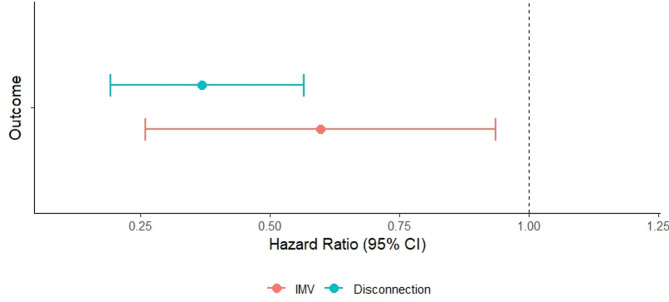
Forest plot representing the association between repeated instances of self-reported dyspnea and both the weaning duration and weaning success. Dyspnea presence was evaluated daily during invasive mechanical ventilation and at the end of the disconnection session. The association between dyspnea at the end of the disconnection and longer weaning duration and unsuccessful weaning outcome was the strongest. IMV: invasive mechanical ventilation; CI: confidence interval

To evaluate the association between dyspnea and weaning outcome at 28 days after initiation of weaning with tracheostomy, three joint model versions were constructed with dyspnea considered during IMV, at the end of disconnection and during IMV and/or at the end of disconnection sessions. In all model versions a history of dyspnea was significantly inversely associated with weaning duration and success (Fig. 2, Table S5). The association between dyspnea at the end of the disconnection session and successful weaning within 28 days was the strongest (mean − 1.00, *P* < 0.001, corresponding with a hazard ratio of 0.37, 95% confidence interval (CI) 0.19; 0.57).

### Association between dyspnea and psychological outcome and health-related QoL

Out of 151 communicative patients, 109 (72%) were alive at 90 days after ICU discharge (Figure S5), and received questionnaires to assess their post-ICU psychological outcome and HR-QoL. Of those, 54 (49%) patients completed the questionnaires. Baseline and dyspnea characteristics did not differ between responders and non-responders (Table S6). Among the responding participants 18 (34%) had probable PTSD, 24 (45%) had probable anxiety disorder and 23 (43%) had probable depression disorder.

**Fig. 3:**
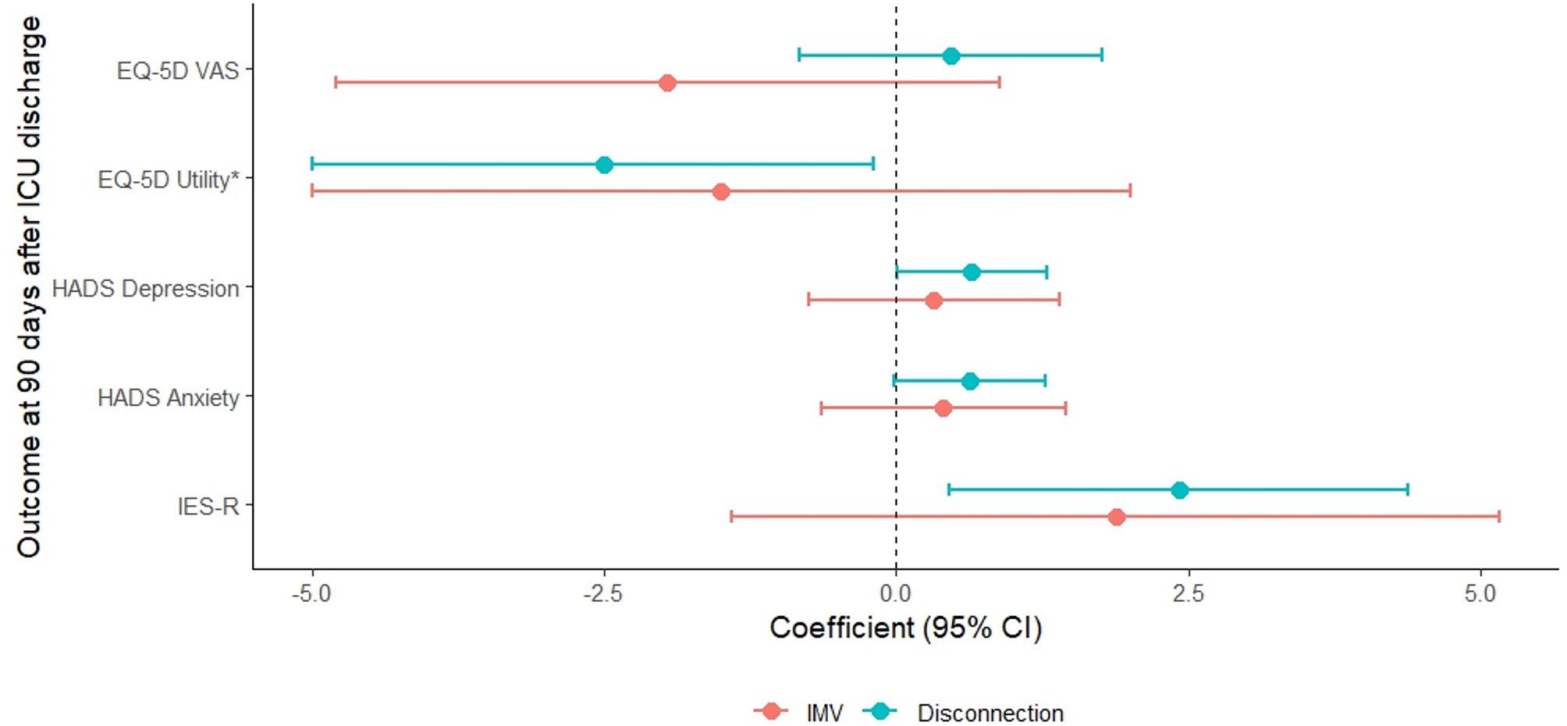
Visual representation of the regression models for associations between dyspnea, weaning duration and post-ICU psychological outcomes at 90 days after ICU discharge. Linear regression was applied for continuous scores. The effect size for linear regressions represents the beta coefficient with confidence interval. ICU: intensive care unit, CI: confidence interval, IES-R: Impact of Event Scale-Revised, HADS: hospital anxiety and depression, EQ-5D: European Quality of life – 5 dimensions, VAS: visual analog scale. *For the EQ-5D utility score, the regression coefficient and 95%CI are expressed per 0.01-unit change (i.e. multiplied by 100) to improve visual clarity. The scaling does not affect statistical significance

To assess the association between dyspnea and 90-day mortality, presence or severity of PTSD, anxiety, depression, and HR-QoL logistic and linear regression analyses were performed, while adjusting for the number of weaning days patients were able to communicate. The cumulative number of weaning days with dyspnea during disconnection was significantly associated with IES-R total score at 90 days (regression coefficient (β) 2.42; 95%CI 0.46; 4.38, *P* = 0.020) and EQ-5D utility score (−0.025; 95%CI −0.050; −0.002, *P* = 0.031, Fig. 3, Table S7). There was no association with 90-day mortality (odds ratio 0.90, 95%CI 0.72; 1.11, *P* = 0.34).

**Table 1:** Baseline characteristics and clinical outcomes of included patients

	Overall (*n* = 156)
Males, n (%)	99 (64)
Age (y)	62 (13)
Body mass index (kg/m^2^)	27.5 (5.7)
Chronic respiratory disease, n (%)	41 (26)
Chronic cardiovascular disease, n (%)	45 (29)
Psychiatric history, n (%)	33 (21)
Charlson Comorbidity Inde**x**	3 [2; 5]
Clinical Frailty Scale at hospital admission	3 [2; 4]
APACHE-IV score at ICU admission	75 [60; 96]
IMV indication at initial intubation, n (%)	
Securing airway	9 (6)
Hypoxemic respiratory failure	58 (37)
Hypercapnic respiratory failure	16 (10)
Hemodynamic	24 (15)
Neurologic	6 (4)
Post-surgery or trauma	43 (28)
Tracheostomy Indication, n (%)	
Long (expected) weaning duration	60 (39)
ICU-acquired weakness	71 (46)
Sputum retention	24 (15)
Other	1 (1)
SOFA score at first disconnection session	6 [4; 9]
PaO_2_/FiO_2_ at first disconnection session	248 [210; 309]
Effective cough effectiveness at first disconnection session	56 (36)
MRC sum score at first disconnection session	27 [12; 40]
Outcome at 28 days after first disconnection session, n (%)	
Successfully weaned from IMV	124 (80)
Still weaning	10 (6)
Died	22 (14)
Outcome at ICU discharge, n (%)	
Successfully weaned, tracheostomy removed	105 (67)
Successfully weaned, tracheostomy in situ	25 (16)
Died	26 (17)
Weaning duration (d)	10 [8; 15]
ICU length of stay (d)	37 [27; 46]
Duration tracheostomy in situ (d)	18 [13; 28]
Hospital length of stay (d)	53 [42; 75]
90-day mortality rate, n (%)	47 (30)

C﻿ategorical variables are presented as number with percentage between brackets. Continuous variables are presented as number ± SD between brackets or quartiles between square brackets, depending on the distribution. Cardiovascular disease included congestive heart failure, myocardial infarction, peripheral arterial disease and stroke. Respiratory disease included chronic obstructive pulmonary disease, asthma and obstructive sleep apnea. Psychiatric history included depression, substance abuse, anxiety, post-traumatic stress and personality disorder. APACHE: acute physiology and chronic health evaluation, ICU: intensive care unit, IMV: invasive mechanical ventilation, MRC: Medical Research Council, SOFA: sequential organ failure assessment

**Table 2:** Sum of available dyspnea assessments on communicative weaning days

	**Yes**	**No**	**NA**
*Dyspnea reported (n, %)*			
During IMV	171 (15)	880 (78)	71 (6)
At end of disconnection session	300 (26)	723 (64)	99 (9)
During IMV or at end of disconnection session	370 (33)	720 (64)	32 (3)
*D-VAS > 3.0 (n,* * %)*			
During IMV	222 (20)	555 (50)	345 (31)
At end of disconnection session	308 (28)	451 (40)	363 (32)
During IMV or at end of disconnection session*	378 (34)	365 (33)	379 (34)

Summary of all dyspnea assessments on communicative weaning days from all patients combined, and represented as absolute numbers with percentages between brackets. These numbers concern repeated measures, i.e. the contribution of included patient differs per patient. *The highest score of the two time-points. NA: not available, IMV: invasive mechanical ventilation, D-VAS: dyspnea visual analog scale

**Table 3:** Summary of dyspnea assessments

	Overall (*n* = 151)
Number of communicative weaning days	7 [4; 12]
*Dyspnea Prevalence (n*,* %)*^±^	88 (58)
*Dyspnea Cumulative incidence (days)* ^±^	
During IMV	2 [1; 3]
At end of disconnection session	2 [1; 5]
During IMV and/or at end of disconnection session	3 [2; 6]
*Dyspnea Density (%)* ^±^	
During IMV	0 [0; 29]
At end of disconnection session	17 [0; 40]
During IMV and/or at end of disconnection session	22 [0; 50]
*Dyspnea Severity (D-VAS)*	
During IMV	
Dyspnea reported: Yes	5 [4; 7]
Dyspnea reported: No	0 [0; 2]
At end of disconnection session	
Dyspnea reported: Yes	6 [4; 7]
Dyspnea reported: No	0 [0; 3]
During IMV and/or at end of disconnection session*	
Dyspnea reported: Yes	6 [5; 7]
Dyspnea reported: No	0 [0; 3]

Dyspnea in communicative patients (i.e. excluding *n* = 5 patients who were noncommunicative during all assessments in the weaning phase). Categorical variables are presented as number with percentage between brackets. Continuous variables are presented as number with SD between brackets or quartiles between square brackets, depending on the distribution. These numbers are the results of a repeated measure approach to account for within-subject correlation and unequal observation counts. ^±^ Dyspnea was reported based on the binary question. *The highest score of the two time-points. Abbreviations: IMV: invasive mechanical ventilation, D-VAS: dyspnea visual analog scale

## Discussion

While recognizing the importance of other patient-centered outcomes like thirst and pain, the current study focused on dyspnea [17]. Dyspnea is increasingly recognized as a clinical and research priority in critically ill patients [10, 13]. This prospective multicenter study presents the first comprehensive evaluation of the prevalence and severity of dyspnea in patients weaning with a tracheostomy. The key findings were the following: first, moderately severe dyspnea was observed on multiple days of the weaning process in a majority of patients, with reported dyspnea present on average on 3 out of 7 weaning days. Second, an increasing number of episodes of dyspnea during weaning with tracheostomy was associated with prolonged weaning duration and reduced weaning success. Moreover, dyspnea especially during disconnection sessions was associated with adverse long-term weaning outcomes, like PTSD symptoms and lower HR-QoL at 90 days after ICU discharge. Finally, the assessment of dyspnea presence with a binary question was more feasible than assessment of dyspnea severity by D-VAS.

This was the first study to prospectively and repeatedly assess dyspnea throughout the tracheostomized weaning phase, offering a detailed account of the burden and consequences of dyspnea during this critical phase. This study confirmed that tracheostomized patients weaning from IMV are at risk for dyspnea, most frequently at the end of disconnection sessions, presumably due to the increased respiratory effort required by progressively extended periods without ventilatory support [13, 33–35]. The prevalence of dyspnea reported was similar or higher than earlier prospective studies in patient on IMV with indwelling endotracheal tubes [6, 13, 17, 36, 37] with reported dyspnea severity levels also exceeding previous studies [6, 17, 36, 37].

A history of dyspnea during tracheostomized weaning is associated with longer weaning duration and lower probability of successful weaning outcome. Dyspnea might be a symptom indicative of underlying disease responsible for impaired outcomes, but increased weaning duration might also reflect a response from healthcare professionals to end disconnection sessions when dyspnea was reported. Furthermore, even after correcting for the weaning duration, the cumulative incidence of dyspnea was associated with the severity of PTSD-symptoms and HR-QoL, aligning with previous hypotheses [10, 33, 38, 39] and confirming an earlier observation in patients during IMV via endotracheal tube [6]. Prolonged weaning exposed patients to repeated dyspnea episodes, which may explain the high incidence of probable PTSD, among the highest measured in ICU survivors [40, 41] or patients on IMV [6]. A clear direction of results suggested an association between the cumulative dyspnea incidence and both anxiety and depression. Collectively, critically ill patients are at particular risk to experience dyspnea during tracheostomized weaning, and the number of dyspnea episodes is associated with weaning outcome and post-ICU psychological sequelae.

The prospective study design with daily repeated measures allowed the exploration of the feasibility of dyspnea assessment in patients weaning with tracheostomy. Unlike previous studies that excluded patients unable to communicate upfront [6, 17], this study prospectively assessed patients’ ability to communicate and presence dyspnea on a daily basis. As a pragmatic study, systematic dyspnea evaluation was not routine practice across all centers, highlighting the feasibility of such assessments in settings lacking specific expertise in dyspnea research. Dyspnea assessment was frequently hindered by patients’ non-communicative states, and even during periods when patients could communicate, data on dyspnea were sometimes missing, especially on dyspnea severity using the D-VAS. This may result from barriers in dyspnea assessments, which may be caretaker-driven (time consuming, not part of routine) or patient-driven (variable cognition within the set boundaries for communicativeness) [13, 43]. Clinicians tend to underestimate the presence and severity of dyspnea [36, 42, 43], emphasizing the need for objective surrogate measures of dyspnea severity that are independent of a patient’s ability to communicate. Currently available hetero-observational scores are promising but lack validating data for clinical practice [13, 37, 44–46]. While the D-VAS remains the preferred method due to its granularity and association with outcomes [13, 47–49], its clinical application in tracheostomized patients appears limited. Our findings suggest that in tracheostomized patients a simpler binary dyspnea assessment is more feasible while maintaining its relevance and association with weaning outcomes.

### Clinical implications

Overall, clinical outcomes in this contemporary cohort of patients with tracheostomized weaning were poor with prolonged weaning duration, high mortality rates, and a high prevalence of post-ICU psychological disorders, confirming earlier studies that patients with prolonged weaning are clearly at risk for poor outcomes. Those outcomes were preceded by a long duration of ICU- and hospital admission, imposing significant costs by utilizing ICU capacity and other healthcare resources [4, 5, 50]. Moreover, the observed association between cumulative incidence of dyspnea during disconnection sessions and impaired post-ICU psychological outcomes and HR-QoL, along with prior evidence linking prolonged weaning to PTSD and depression [34, 51] supports the need for daily dyspnea assessment during weaning. This also advocates future research on interventions to prevent or treat dyspnea during tracheostomized weaning, especially during disconnection sessions. Several pharmacologic and non-pharmacologic interventions have potential to ameliorate dyspnea in patients on IMV [13, 52]. It remains to be investigated whether dyspnea reflects underlying disease or respiratory muscle weakness, or the symptom itself has a causal relationship with outcome. Causal inference could be investigated in randomized studies evaluating whether interventions aimed to relieve dyspnea during weaning translate into improved clinical outcomes. The current study establishes baseline data on dyspnea that may serve as foundation for such future studies in tracheostomized patients weaning from IMV.

### Strengths and limitations

Strengths of this study include a thorough dyspnea assessment and generalizability through the performance of a prospective, pragmatic multicenter study on a distinct at-risk population. Analyses accounting for repeated measurements allowed for detailed evaluation of the burden and consequences of dyspnea and the feasibility of dyspnea assessment in ventilated critically ill patients. However, there are also limitations to consider. First, an association between dyspnea, weaning duration and weaning outcomes was found but its mechanism remains undefined. Whether dyspnea is a symptom indicative of underlying disease or directly responsible for impaired outcomes remains to be investigated. Randomized studies evaluating whether interventions that relieve dyspnea also translate into improved clinical outcomes are required. Second, this study was conducted in Dutch ICUs who predominantly used protocolized disconnection sessions as strategy to wean patients. Whether our results can be generalized to other weaning strategies is unclear [53]. Daily weaning management was left at the discretion of the treating clinical team as high level evidence to guide weaning is largely lacking. However, the similarity of weaning approach in this large multicenter study, the multicenter nature of this study, the frequent use of disconnection session strategies elsewhere [18] and unassisted breathing being a core element of weaning, suggest that the direction of results will likely be similar. Third, weaning strategies and interventions that may affect dyspnea sensation were not standardized, which may have introduced modest inter-center heterogeneity and make direct causal reasoning hazardous. However, this variability is inherent to contemporary weaning practice and the real-world data of this study can be of guidance for the design of future pragmatic interventional studies on dyspnea during weaning with tracheostomy. Fourth, the analysis on post-ICU psychological outcomes had a relatively small sample size and may be hampered by response bias. However, response bias is unlikely to have greatly affected the results on psychological recovery, as non-responders had similar characteristics as responders, especially cumulative dyspnea incidence. Lastly, data on the descriptor of dyspnea such as air hunger, excessive effort and chest constriction were not collected [54]. Such differentiation could be of guidance for pathophysiological studies into dyspnea [55], but more in-depth assessment was deemed unfeasible in our study.

## Conclusion

Tracheostomized critically ill patients weaning from IMV frequently experience dyspnea, particularly during disconnection sessions. Dyspnea is associated with prolonged weaning duration, reduced weaning success, more PTSD symptomatology and lower health-related quality of life post-ICU. These findings underscore the importance for healthcare workers of recognizing and addressing the consequences of dyspnea in these patients. Giving the significant influence of dyspnea in patients weaning with a tracheostomy, future pragmatic studies should target alleviating dyspnea as assessed by a simple tool, with the aim of improving clinical outcome.

### Take home message

The burden of dyspnea during the weaning phase from mechanical ventilation with tracheostomy is high. Dyspnea is associated with longer weaning duration and adverse short- and long term outcomes.

### Tweet

The burden of dyspnea during tracheostomized weaning is high, and is associated with adverse weaning outcomes. 

## Supplementary Information

Supplementary Material 1.

## Data Availability

The datasets used and/or analysed during the current study are available from the corresponding author on reasonable request.

## Abbreviations

APACHE: Acute physiology and chronic health evaluation
CAM-ICU: Confusion assessment method for the Intensive Care Unit
CI: Confidence interval
D-VAS: Dyspnea visual analog scale
ECCO2R: Extracorporeal carbon dioxide removal
ECMO: Extracorporeal membrane oxygenation
EQ-5D: EuroQol 5 dimensions
EQ-VAS: EuroQol visual analog scale
HADS: Hospital anxiety and depression scale
HR: Hazard Ratio
HR-QoL: Health-related quality of life
ICDSC: Intensive care delirium screening checklist
ICU: Intensive care unit
IES-R: Impact of events – revised
IMV: Invasive mechanical ventilation
MRC: Medical research council
NA: Not available
PTSD: Post-traumatic stress disorder
SD: Standard deviation
SOFA: Sequential organ failure assessment
