# Supplementary material for: Dyspnea is related to clinical outcomes in patients weaning from invasive mechanical ventilation with tracheostomy: a multicenter prospective study
Source: Crit Care. 2026 Jan 8;30:16. doi: 10.1186/s13054-025-05734-8 (PMC12784585; doi:10.1186/s13054-025-05734-8)
Supplement: Supplementary file 1 — Supplementary Material 1. [file 13054_2025_5734_MOESM1_ESM.docx]

**Supplementary material**

**Dyspnea is related to Clinical Outcomes in Patients Weaning from Invasive Mechanical Ventilation with Tracheostomy: a multicenter prospective study**

M.L. Janssen^1,2,3^, H. Endeman^2,4^, Z. Yang^5^, J.H. Elderman^2,6^, M. Goeijenbier^2,7^, T. Dongelmans^7^, H. Moeniralam^8^, J. Rozendaal^8^, A.J.A.M. van Hees^9^, J.D. Workum^2,9^, E.A.N. Oostdijk^10^, P. Petersen^10^, D. van Nieuwenhuizen^11^, T. van Zuylen^11^, A. De Bie Dekker^12,13^, I. Herold^12,13^, S. Stads^14^, S. Achterberg^15^, A. Osinski^16^, L. Heunks^2,17^, E-J. Wils^1,2^, on behalf of the Trach-Wean study group.

1. Intensive Care, Franciscus Gasthuis & Vlietland, Rotterdam,
2. Intensive Care, Erasmus MC, Rotterdam,
3. Pulmonary Medicine, Erasmus MC, Rotterdam,
4. Intensive Care, OLVG, Amsterdam
5. Department of Epidemiology and Biostatistics, Erasmus University Medical Center
6. Intensive Care, IJsselland Ziekenhuis, Capelle a/d IJssel,
7. Intensive Care, Spaarne Gasthuis, Haarlem
8. Intensive Care, Sint Antonius Ziekenhuis, Nieuwegein
9. Intensive Care, Elizabeth-Tweesteden Ziekenhuis, Tilburg
10. Intensive Care, Rijnstate Ziekenhuis, Arnhem
11. Intensive Care, Jeroen Bosch Ziekenhuis, ’s Hertogenbosch
12. Intensive Care, Catharina Ziekenhuis, Eindhoven
13. Intensive Care, Anna Ziekenhuis, Geldrop
14. Intensive Care, Ikazia Ziekenhuis, Rotterdam
15. Intensive Care, HMC Westeinde, Den Haag
16. Intensive Care, Maxima Medisch Centrum, Veldhoven
17. Intensive Care, Radboud UMC Nijmegen

**Collaborators:** Annika Dingenouts, Julia Verwaaijen, Stefan Ras, Manouk van Oevelen, Elbert Bot, Stefan Ras, Arie Meijer, Sjoerd Stok, Robin Leupe, M. Cornelisse A.G. Sybesma-Prins, Jeannette Middendorp, Jeroen van Rosmalen and Koen Simons.

**Corresponding author**

Evert-Jan Wils MD PhD

Department of Intensive Care, Franciscus Gasthuis & Vlietland

Kleiweg 500, 3045 PM Rotterdam, the Netherlands

E-mail: e.wils@franciscus.nl

Telephone: 0031-(0)10-4616161

Content

[1. STROBE Statement—checklist 3](#_Toc192249651)

[2. Study setting 5](#_Toc192249652)

[3. Data collection 8](#_Toc192249653)

[4. Data analysis 13](#_Toc192249654)

[5. Supplemental data 15](#_Toc192249655)

[6. References 20](#_Toc192249656)

### STROBE Statement—checklist

|  | Item No | Recommendation | Page n^o^ |
| --- | --- | --- | --- |
| **Title and abstract** | 1 | (*a*) Indicate the study’s design with a commonly used term in the title or the abstract | 1 |
|  |  | (*b*) Provide in the abstract an informative and balanced summary of what was done and what was found | 4-5 |
| Introduction | | |  |
| Background/rationale | 2 | Explain the scientific background and rationale for the investigation being reported | 6 |
| Objectives | 3 | State specific objectives, including any prespecified hypotheses | 6 |
| Methods | | |  |
| Study design | 4 | Present key elements of study design early in the paper | 7 |
| Setting | 5 | Describe the setting, locations, and relevant dates, including periods of recruitment, exposure, follow-up, and data collection | 7 |
| Participants | 6 | (*a*) *Cohort study*—Give the eligibility criteria, and the sources and methods of selection of participants. Describe methods of follow-up | 7 |
|  |  | (*b*) *Cohort study*—For matched studies, give matching criteria and number of exposed and unexposed | NA |
| Variables | 7 | Clearly define all outcomes, exposures, predictors, potential confounders, and effect modifiers. Give diagnostic criteria, if applicable | 8 |
| Data sources/ measurement | 8* | For each variable of interest, give sources of data and details of methods of assessment (measurement). Describe comparability of assessment methods if there is more than one group | 8,9 |
| Bias | 9 | Describe any efforts to address potential sources of bias | 9, supplements |
| Study size | 10 | Explain how the study size was arrived at | 9 |
| Quantitative variables | 11 | Explain how quantitative variables were handled in the analyses. If applicable, describe which groupings were chosen and why | 9,10 supplements |
| Statistical methods | 12 | (*a*) Describe all statistical methods, including those used to control for confounding | 9,10 supplements |
|  |  | (*b*) Describe any methods used to examine subgroups and interactions | 9, supplements |
|  |  | (*c*) Explain how missing data were addressed | 9, supplements |
|  |  | (*d*) *Cohort study*—If applicable, explain how loss to follow-up was addressed | NA |
|  |  | (*e*) Describe any sensitivity analyses | NA |

| Results | | | Page n^o^ |
| --- | --- | --- | --- |
| Participants | 13* | (a) Report numbers of individuals at each stage of study—eg numbers potentially eligible, examined for eligibility, confirmed eligible, included in the study, completing follow-up, and analysed | 11 |
|  |  | (b) Give reasons for non-participation at each stage | NA |
|  |  | (c) Consider use of a flow diagram | Fig S4 |
| Descriptive data | 14* | (a) Give characteristics of study participants (eg demographic, clinical, social) and information on exposures and potential confounders | 11, 12 |
|  |  | (b) Indicate number of participants with missing data for each variable of interest | 15 |
|  |  | (c) *Cohort study*—Summarise follow-up time (eg, average and total amount) | 13 |
| Outcome data | 15* | *Cohort study*—Report numbers of outcome events or summary measures over time | 11-16 |
| Main results | 16 | (*a*) Give unadjusted estimates and, if applicable, confounder-adjusted estimates and their precision (eg, 95% confidence interval). Make clear which confounders were adjusted for and why they were included | 11-16 |
|  |  | (*b*) Report category boundaries when continuous variables were categorized | 11-16 |
|  |  | (*c*) If relevant, consider translating estimates of relative risk into absolute risk for a meaningful time period | 11-16, Table S5 |
| Other analyses | 17 | Report other analyses done—eg analyses of subgroups and interactions, and sensitivity analyses | Supplements, table S4-6 |
| Discussion | | |  |
| Key results | 18 | Summarise key results with reference to study objectives | 19 |
| Limitations | 19 | Discuss limitations of the study, taking into account sources of potential bias or imprecision. Discuss both direction and magnitude of any potential bias | 21-22 |
| Interpretation | 20 | Give a cautious overall interpretation of results considering objectives, limitations, multiplicity of analyses, results from similar studies, and other relevant evidence | 19-21 |
| Generalisability | 21 | Discuss the generalisability (external validity) of the study results | 22 |
| Other information | | |  |
| Funding | 22 | Give the source of funding and the role of the funders for the present study and, if applicable, for the original study on which the present article is based | 2 |

**Based on the STROBE research council statement [1]**

### Study setting

In all participating centers the general tracheostomized weaning approach consisted of sessions of disconnection from IMV without ventilatory support, which were progressively extended until patients are able to breathe independently. Information on local facilities and weaning practice is provided in **Tables S1 and S2** below.

| **Table S1. Overview of local weaning practice during the study period** | | | | | | | | | |
| --- | --- | --- | --- | --- | --- | --- | --- | --- | --- |
| **Center** | **Daytime nurse-patient ratio** | **Standardized schedule disconnections** | **Disconnection frequency determined upfront** | **Disconnection duration determined upfront** | **Disconnection time undeter-mined, until respiratory failure** | **Standard respiratory support** | **Use of tracheal high-flow oxygen during disconnection + indication** | **Use of speech valve** | **Use of PMV** |
| **1** | 1:1.5 | No | Yes | Yes | No | HME | Yes, hypoxemia on HME | Yes | No |
| **2** | 1:1.4 | Yes | Yes | Yes | No | HME | Yes, hypoxemia on HME | Yes | Yes |
| **3** | 1:1.5 | No | Yes | Yes | No | HME | Yes, hypoxemia on HME | Yes | No |
| **4** | 1:1.5 | Yes | Yes | Yes | No | HME | Yes, hypoxemia on HME | Yes | Yes |
| **5** | 1:1.5 | Yes | Yes | Yes | No | HME | Yes, hypoxemia and obesity | Yes | Yes |
| **6** | 1:2 | Yes | Yes | Yes | No | HME | Yes, copious secretions | Yes | Yes |
| **7** | 1:2 | Yes | Yes | Yes | No | HME/HFO | Yes, both interchangeably | Yes | No |
| **8** | 1:2 | Yes | Yes | Yes | No | HME | Yes, hypoxemia, high effort | Yes | No |
| **9** | 1:1.5 | Yes | Yes | Yes | No | HME | Yes, hypoxemia on HME | Yes | No |
| **10** | 1:1.5 | Yes | Yes | Yes | Yes | HFO | Yes, clinical standard | Yes | No |
| **11** | 1:1.5 | Yes | Yes | Yes | No | HME | Yes, hypoxemia on HME | Yes | No |
| **12** | 1:2 | Yes | Yes | Yes | No | HME/HFO | Yes, both interchangeably | Yes | Yes |
| **13** | 1:1.5 | Yes | Yes | Yes | No | HME | Yes, hypoxemia on HME | Yes | Yes |
| Categorical variables are presented as number with percentage. Abbreviations: HME: Heat-moisture exchanger (i.e. artificial nose), HFO: high-flow oxygen, PMV: Passy-Muir valve | | | | | | | | | |

| **Table S2. Overview of local resources and practices relevant to tracheostomized weaning during the study period** | | | | | | | | | | |
| --- | --- | --- | --- | --- | --- | --- | --- | --- | --- | --- |
| **Center** | **Inclusions** | **ICU capacity** | **Intensivist fte** | **Nurse fte** | **Tracheostomy type** | **Standard tracheostomy tube size** | **Surgical tracheostomy procedure** | **Percutaneous tracheostomy procedure** | **Tracheostomy procedure performance** |  |
| **1** | 2 (1) | 5 | 4 | 21 | Tracoe | 7 | No | Yes | I |  |
| **2** | 11 (7) | 20 | 13 | 86 | Tracoe | 7 female, 8 male | No | Yes | I |  |
| **3** | 4 (23) | 24 | 8 | 79 | Tracoe | 7 female, 8 male | No | Yes | I |  |
| **4** | 59 (38) | 48 | 20 | 182 | Shiley | 6 female, 8 male | Yes | Yes | I, O |  |
| **5** | 11 (7) | 24 | 19 | 93 | Tracoe, Shiley | 7 female, 8 male | Yes | Yes | I, O, S |  |
| **6** | 16 (10) | 16 | 10 | 44 | Tracoe | 8 | Yes | Yes | I, S |  |
| **7** | 4 (3) | 22 | 13 | 61 | Tracoe | 7 female, 8 male | Yes | Yes | I, O, S |  |
| **8** | 4 (3) | 8 | 6 | 28 | Shiley | 8 | Yes | Yes | I, S |  |
| **9** | 7 (5) | 16 | 7 | 61 | Tracoe | 8 | Yes | Yes | I, S |  |
| **10** | 3 (2) | 13 | 7 | 48 | Tracoe | 7 female, 8 male | Yes | Yes | I, S |  |
| **11** | 10 (6) | 13 | 8 | 63 | Tracoe | 7 female, 8 male | No | Yes | **I** |  |
| **12** | 10 (6) | 16 | 11 | 53 | Tracoe | 7 female, 8 male | No | Yes | **I, S** |  |
| **13** | 15 (10) | 8 | 5 | 33 | Tracoe | 7 female, 8 male | No | Yes | **I** |  |
| Categorical variables are presented as number with percentage. Abbreviations: fte: full-time equivalent.  Performance tracheostomy procedure: I = Intensivist, O = Otorhinolaryngologist, S = Surgeon, A = Anesthesiologist | | | | | | | | | | |

### Data collection

Each participating center was instructed on the method of dyspnea assessment prior to the start of the study. First, two meetings with all local principal researchers were organized on the aim and general outline of the study. Then, after obtaining local approval, a site-specific initiation visit was performed at each research site with all local research staff involved in the study. Here, all research staff was instructed on which data to collect and how to perform the required study procedures. Specifically, the in- and exclusion criteria, clinical data collection and especially the dyspnea assessment (see below) were explained in detail.

Data were collected at pre-specified time-points: upon inclusion, at start of tracheostomized weaning (i.e. the first disconnection session with tracheostomy), and daily prior to and at the end of the disconnection sessions during the first 28 days of the tracheostomized weaning phase, at ICU- and hospital discharge, and 3 months after ICU discharge (**Figure S1**). In case of multiple disconnection sessions on one day, data were preferentially collected during the first disconnection of the day. Daily data collection was continued until patients were breathing without ventilatory support for 24 hours consecutively, ICU discharge, or 28 days after the initial disconnection session. In case of reconnection to IMV within 7 days after breathing independently for 24 hours, daily data collection was restarted and continued up to 28 days after the initial disconnection session (**Figure S2**). Reconnection to IMV after 7 consecutive days of breathing without ventilatory support or after ICU after discharge were considered as a new event during which dyspnea and other study data were no longer collected on a daily basis.

**Figure S1** Data collection points. Abbreviations: IMV: invasive mechanical ventilation, MRC: medical research council, CAM-ICU: Confusion Assessment Method for the Intensive Care Unit, ICDSC: Intensive Care Delirium Screening Checklist, VAS: visual analog scale, RASS: Richmond agitation-sedation scale, EQ-5D-5L:European Quality of Life, 5D: five-dimensions, 5L: five-level, IES-R: Impact of Event Scale-Revised, HADS: Hospital Anxiety and Depression Scale

*Dyspnea assessment*

Self-reported dyspnea was evaluated as described by others [2-4], and is depicted in **Figure S2**. It was first determined whether a patient was sufficiently able to communicate. The ability to communicate was operationalized as [2-5]: a negative delirium screening (negative Confusion Assessment Method for the Intensive Care Unit (CAM-ICU) or Intensive Care Delirium Screening Checklist (ICDSC) score <4) [6, 7], and a score between -2 and +2 on the Richmond Agitation and Sedation Scale (RASS) [8]. Patients who were considered non-communicative during the full 28-day study period of tracheostomized weaning, were excluded from analyses on dyspnea assessments and the association between dyspnea and outcomes.


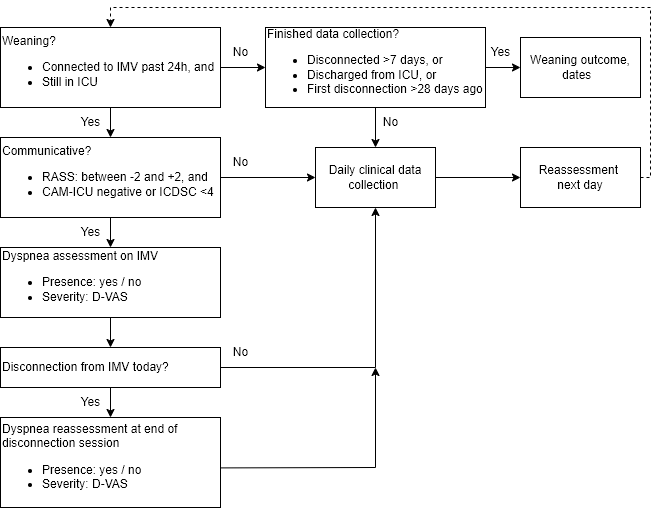


**Figure S2.** Flowchart of daily data collection flowchart during the first 28 days of the tracheostomized weaning period. Abbreviations: CAM-ICU: Confusion Assessment Method for the Intensive Care Unit, ICDSC: Intensive Care Delirium Screening Checklist, RASS: Richmond Agitation and Sedation Scale, D-VAS: dyspnea visual analog scale, IMV: invasive mechanical ventilation, ICU: intensive care unit.

In communicative patients, the presence of dyspnea was assessed daily prior to and at the end of disconnection sessions. The presence of dyspnea was assessed using a binary question (‘Do you experience shortness of breath or trouble breathing at this moment?’). The answer was verified by asking whether if the perceived presence or absence was correctly interpreted by the researcher. Presence of dyspnea was defined as an affirmative answer to this binary question and congruent confirmation; consequently, a day with dyspnea was defined as presence of dyspnea prior to during disconnection.

Regardless of the answer to the binary dyspnea question, patients were asked to rate their dyspnea severity by pointing a score on a visual analogue scale (D-VAS**; Figure S3**) [2-4]. At the first assessment, the D-VAS was explained to patients (score 0= no dyspnea, score 10= worst imaginable dyspnea, i.e. having the feeling to suffocate), and if necessary the explanation was repeated at following assessments. When patients were unable to point to the D-VAS, dyspnea severity was evaluated by moving a pen pointed at the D-VAS from 0 (no dyspnea) to 10 (worst imaginable dyspnea, having the feeling to suffocate) which was stopped when patients indicated to stop the moving pen. Thereafter, the final score was verified by asking the patients if the severity was perceived correctly. If necessary, this approach was repeated until congruent answers were obtained.


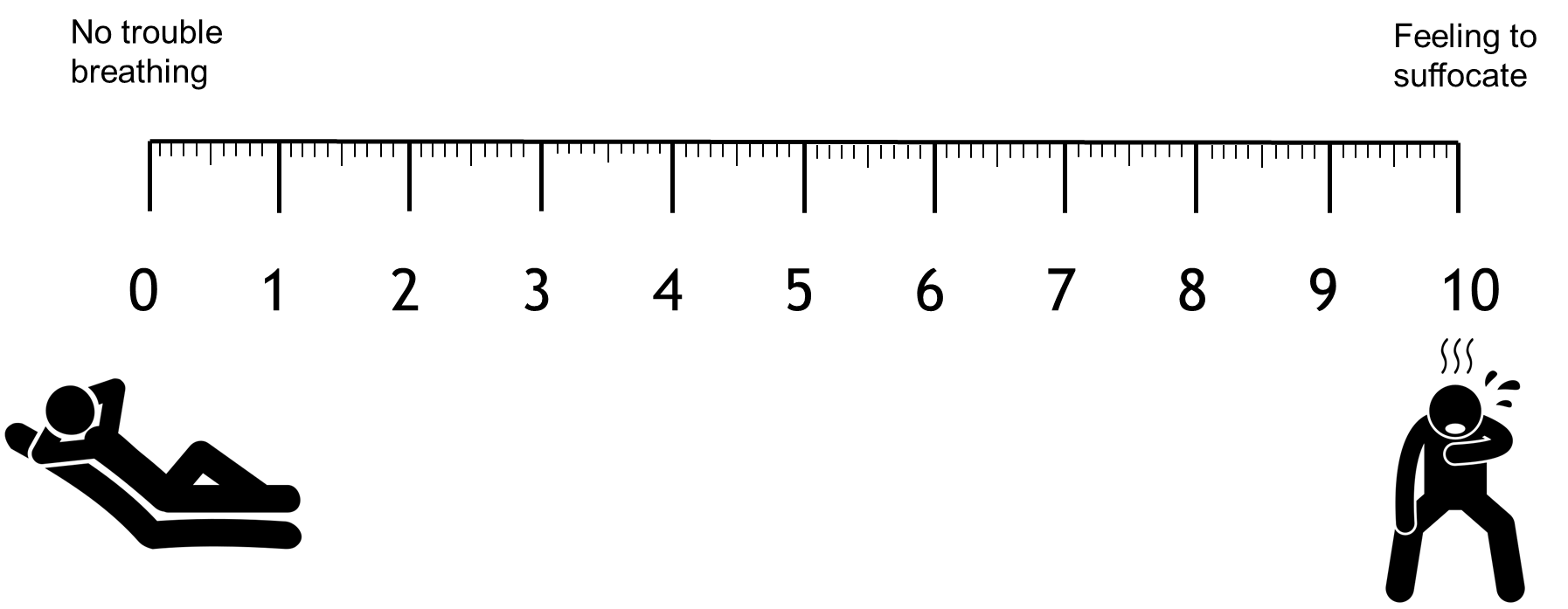


**Figure S3. Dyspnea Visual analog scale (D-VAS)**

*Psychological outcome and health-related QoL after ICU discharge*

Patients were followed up for 90 days after ICU discharge. At 90 days after ICU discharge, HR-QoL and presence of psychological sequelae (anxiety, depression and post-traumatic stress) and severity of psychological symptoms were assessed using validated online questionnaires. Questionnaires were sent through e-mail and completed online. Patients received a reminder twice after two and four weeks in case of no response. The Impact of Event Scale-Revised (IES-R) questionnaire was used to evaluate symptoms associated with post-traumatic stress disorder (PTSD): intrusion, avoidance, and hyperarousal [9, 10]. The IES-R sum score is a measure for the severity of PTSD symptoms, and a IES-R sum score equal or greater than 22 is defined as a probable diagnosis of PTSD. Anxiety and depression were assessed using the Hospital Anxiety and Depression Scale (HADS) questionnaire [11, 12]. A HADS sum score ≥8 on either the depression or anxiety subscale is considered as a probable diagnosis of depression or anxiety, respectively. HR-QoL was assessed using the European Quality of Life 5D (EQ-5D) questionnaire [13, 14] and the European Quality of Life VAS (EQ-VAS). EQ-5D assesses five dimensions (mobility, self-care, usual activities, pain/discomforts, and anxiety/depression) and can be summarized as the EQ-5D utility score, ranging from –0.446 (worst quality of life) to 1.000 (best quality of life). The EQ-VAS corresponds to the patient’s self-reported subjective QoL with a score ranging from 0 (worst health imaginable) to 100 (best health imaginable) provided by patients [15].

### Data analysis

*Association between dyspnea and 28-day weaning outcome*

The association between dyspnea and duration and outcome of weaning was evaluated with joint model analysis. The joint model combines a time-to-event analysis (i.e. Cox proportional hazard model with weaning duration and success) with repeated data analysis (i.e. mixed model with daily dyspnea assessments) while controlling for missing data at random, such as due to non-communicative state [16]. The survival component of the joint model was a Cox model evaluating the weaning time and outcome at 28 days after the start of tracheostomized weaning. The 28-day weaning outcome was weaning success. Success was defined as discharged alive from ICU without ventilator requirement, and/or disconnection for 7 consecutive days. Patients still weaning at 28 days were censored. The survival analysis accounted for the competing risk between death and weaning success as survival outcome. The longitudinal component of the joint model was a generalized linear mixed model with repeated assessments of dyspnea as dependent outcome and time as independent variables, with random intercept and slope. The repeated measure was the daily dyspnea assessment of presence or absence of self-reported dyspnea. The output of the joint model was a single coefficient, which was exponentiated into a Hazard ratio representing the association between repeated instances of dyspnea and both the duration until weaning outcome and successful weaning outcome.

Three different joint models were constructed to analyze the association between dyspnea during IMV, dyspnea during disconnection or dyspnea prior to and/or during disconnection sessions, with weaning outcome. The choice functional form of the joint model for dyspnea was ‘area’, based on the Watanabe–Akaike information criterion value [17]. Joint modeling was performed with the JMbayes2 package as instructed on the website of its developer [18].

*Association between dyspnea and psychological outcome and health-related QoL*

To evaluate associations between the cumulative incidence of dyspnea during weaning and questionnaire outcomes regression analyses were used. Linear regression was applied for continuous scores (IES-R, HADS-a and HADS-d, EQ-5D-TTO, EQ-VAS score). The models assessed the relationship between the dependent variables and the number of days with dyspnea, controlling for weaning duration by including the number of communicative weaning days as an independent variable.

### Supplemental data

| **Table S3. Baseline characteristics and clinical outcomes in patients with low and high dyspnea density** | | | |
| --- | --- | --- | --- |
|  | **Low dyspnea**  **density (n=84)** | **High dyspnea density (n=67)** | **P-value** |
| **Males** | 51 (61) | 45 (67) | 0.517 |
| **Age (y)** | 64 (12) | 60 (14) | 0.105 |
| **Body mass index (kg/m^2^)** | 28.0 (5.9) | 27.0 (5.7) | 0.283 |
| **Chronic respiratory disease** | 19 (23) | 20 (30) | 0.411 |
| **Chronic cardiovascular disease** | 22 (26) | 21 (31) | 0.606 |
| **Psychiatric history** | 17 (20) | 14 (21) | 1 |
| **Charlson Comorbidity Index score** | 3 [2, 4] | 3 [2, 4] | 0.965 |
| **Clinical Frailty Scale at hospital admission** | 3 [2, 3] | 3 [2, 4] | 0.068 |
| **APACHE-IV score at ICU admission** | 75 [62, 95] | 77 [56, 96] | 0.919 |
| **IMV indication at initial intubation** |  |  | 0.765 |
| **Securing airway** | 3 (4) | 5 (8) |  |
| **Hypoxemic respiratory failure** | 28 (33) | 27 (40) |  |
| **Hypercapnic respiratory failure** | 10 (12) | 6 (9) |  |
| **Hemodynamic** | 13 (16) | 10 (15) |  |
| **Neurologic** | 4 (5) | 2 (3) |  |
| **Post-surgery or trauma** | 26 (31) | 17 (25) |  |
| **Tracheostomy Indication** |  |  | 0.508 |
| **Long (expected) weaning duration** | 34 (41) | 23 (34) |  |
| **ICU-acquired weakness** | 35 (42) | 35 (52) |  |
| **Sputum retention** | 14 (17) | 9 (13) |  |
| **Other** | 1 (1) | 0 |  |
| **Time IMV at first disconnection session** | 15 [9, 18] | 14 [11, 21] | 0.655 |
| **SOFA score at first disconnection session** | 6 [4, 9] | 6 [4, 10] | 0.778 |
| **PaO_2_/FiO_2_ at first disconnection session** | 261 [221, 309] | 246 [210, 310] | 0.614 |
| **pH at first disconnection session** | 7.45 (0.05) | 7.46 (0.05) | 0.428 |
| **Pressure Support level at start weaning (cm H_2_O)** | 8 [6, 10] | 10 [8, 12] | 0.012 |
| **Respiratory rate on ventilator at first disconnection session (breaths/min)** | 21 [18, 25] | 21 [18, 25] | 0.784 |
| **Tidal volume on ventilator at first disconnection session (mL)** | 512 (133) | 502 (124) | 0.622 |
| **Effective cough effectiveness at first disconnection session** | 29 (35) | 25 (37) | 0.897 |
| **MRC sum score at first disconnection session** | 27 [11, 36] | 26 [12, 41] | 0.473 |
| **Weaning duration (d)** | 8 [7; 12] | 13 [9; 22] | <0.001 |
| **Outcome at 28 days after first disconnection session, n (%)** |  |  | 0.001 |
| **Successfully weaned from IMV** | 70 (83) | 50 (75) |  |
| **Still weaning** | 14 (17) | 7 (10) |  |
| **Died** | 0 | 10 (15) |  |
| **90-day mortality rate, n (%)** | 23 (27) | 20 (30) | 0.879 |

Patients were stratified by dyspnea density, based on the median = 22% of communicative weaning days with dyspnea. Patient with dyspnea density below the median were stratified into the low dyspnea density group, and patients with density above the median were stratified into the high dyspnea density group. Categorical variables are presented as number with percentage between brackets. Continuous variables are presented as number with SD between brackets or quartiles between square brackets, depending on the distribution. Between-group comparisons were evaluated with χ2 or Fisher exact test (categorical variables) or the student t-test or Mann-Whitney U test, as appropriate (continuous variables). Cardiovascular disease included congestive heart failure, myocardial infarction, peripheral arterial disease and stroke. Respiratory disease included chronic obstructive pulmonary disease, asthma and obstructive sleep apnea. Psychiatric history included depression, substance abuse, anxiety, post-traumatic stress and personality disorder. Abbreviations: APACHE: acute physiology and chronic health evaluation, ICU: intensive care unit, IMV: invasive mechanical ventilation, SOFA: sequential organ failure assessment, MRC: Medical Research Council.

| **Table S4: Association between daily dyspnea, weaning duration and weaning outcome** | | | |
| --- | --- | --- | --- |
|  | **Dyspnea estimated mean** | **HR (95%CI)** | **P-value** |
| **IMV** | -0.514 | 0.641 (0.320; 0.978) | 0.037 |
| **Disconnection** | -0.997 | 0.369 (0.191; 0.566) | <0.001 |
| **IMV and / or disconnection** | -0.759 | 0.468 (0.299; 0.656) | <0.001 |

Results based on joint models calculating the hazard for successful weaning, while accounting for the competing risk of death. Methods described in supplemental information on data collection and analysis. Abbreviations: IMV: invasive mechanical ventilation, HR: hazard ratio, CI confidence interval

**Figure S4. Study flowchart**

**
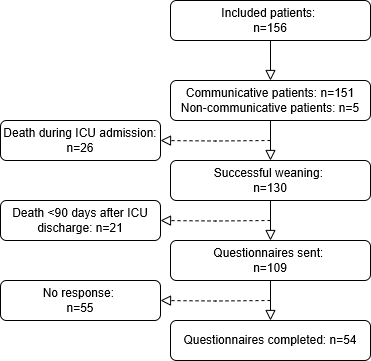
**

| **Table S5. Baseline characteristics and clinical outcomes in non-responders and responders to the questionnaires at day 90 post-ICI discharge** | | | |
| --- | --- | --- | --- |
|  | **Non-responders (n=55)** | **Responders (n=54)** | **P-value** |
| **Males** | 36 (65.5) | 34 (63) | 0.943 |
| **Age (y)** | 59 (17) | 62 (11) | 0.312 |
| **Body mass index (kg/m^2^)** | 27 (5) | 29 (6) | 0.148 |
| **Chronic respiratory disease** | 14 (26) | 16 (30) | 0.784 |
| **Chronic cardiovascular disease** | 15 (27) | 10 (19) | 0.390 |
| **Psychiatric history** | 10 (18) | 14 (26) | 0.457 |
| **Clinical Frailty Scale at hospital admission** | 3 [2; 4] | 3 [2; 3] | 0.957 |
| **APACHE-IV score at ICU admission** | 70 [55; 96] | 74 [58; 89] | 0.956 |
| **Charlson Comorbidity Index score** | 3 [2; 4] | 3 [2; 4] | 0.713 |
| **Tracheostomy Indication** |  |  | 0.059 |
| **Long (expected) weaning duration** | 16 (29) | 23 (43) |  |
| **ICU-acquired weakness** | 23 (42) | 26 (48) |  |
| **Sputum retention** | 15 (27) | 5 (9) |  |
| **Other** | 1 (2) | 0 |  |
| **MRC sum score at start weaning** | 28 [14; 40] | 29 [14; 44] | 0.580 |
| **Number of suctioning procedures per day** | 7 [5; 8] | 5 [4; 8] | 0.051 |
| **Dyspnea cumulative incidence (days)** | 2 [1; 6] | 3 [2; 6] | 0.343 |
| **Dyspnea density (%)** | 19 [0; 43] | 24 [0; 53] | 0.321 |
| **IMV until tracheostomy (d)** | 14 [8; 20] | 15 [12, 21] | 0.088 |
| **IMV with tracheostomy until start weaning (d)** | 1 [1, 2] | 1 [1, 2] | 0.812 |
| **Weaning duration (d)** | 9 [8; 12] | 9 [8; 15] | 0.362 |
| **Total IMV duration (d)** | 25 [20, 32] | 31 [23, 37] | 0.029 |
| **ICU length of stay (d)** | 34 [26; 40] | 38 [30; 46] | 0.133 |
| **Hospital length of stay (d)** | 55 [42; 77] | 56 [46; 73] | 0.857 |

Categorical variables are presented as number with percentage between brackets. Continuous variables are presented as number with SD between brackets or quartiles between square brackets, depending on the distribution. Between-group comparisons were evaluated with χ2 or Fisher exact test (categorical variables) or the student t-test or Mann-Whitney U test, as appropriate (continuous variables). Cardiovascular disease included congestive heart failure, myocardial infarction, peripheral arterial disease and stroke. Respiratory disease included chronic obstructive pulmonary disease, asthma and obstructive sleep apnea. Psychiatric history included depression, substance abuse, anxiety, post-traumatic stress and personality disorder. Abbreviations: APACHE: acute physiology and chronic health evaluation, ICU: intensive care unit, IMV: invasive mechanical ventilation, MRC: Medical Research Council.

| **Table S6. Association between dyspnea and psychological symptoms and HR-QoL at 90 days post ICU-discharge** | | | | |
| --- | --- | --- | --- | --- |
| **Outcome** | **Dyspnea** | **Coefficient** | **95%CI** | **P-value** |
| **IES-R** | IMV | 1.88 | -1.41; 5.17 | 0.250 |
|  | Disconnection | 2.42 | 0.46; 4.38 | 0.020 |
|  | IMV and/or disconnection | 2.27 | 0.58; 3.96 | 0.010 |
|  |  |  |  |  |
| **HADS anxiety** | IMV | 0.40 | -0.64; 1.45 | 0.431 |
|  | Disconnection | 0.62 | -0.02; 1.28 | 0.057 |
|  | IMV and/or disconnection | 0.59 | -0.02; 1.19 | 0.060 |
|  |  |  |  |  |
| **HADS depression** | IMV | 0.33 | -0.749; 1.40 | 0.535 |
|  | Disconnection | 0.65 | 0.002; 1.29 | 0.049 |
|  | IMV and/or disconnection | 0.61 | -0.02; 1.24 | 0.060 |
|  |  |  |  |  |
| **EQ-5D utility score** | IMV | -0.02 | -0.05; 0.02 | 0.409 |
|  | Disconnection | -0.03 | -0.05; -0.002 | 0.031 |
|  | IMV and/or disconnection | -0.19 | -0.04 ; 0.00 | 0.070 |
|  |  |  |  |  |
| **EQ-5D VAS** | IMV | -1.96 | -4.80; 0.88 | 0.165 |
|  | Disconnection | 0.47 | -0.83; 1.77 | 0.448 |
|  | IMV and/or disconnection | -0.50 | -2.44; 1.45 | 0.610 |

Regression models for associations between dyspnea, weaning duration and post-ICU psychological outcomes at 90 days after ICU discharge. Linear regression was applied for continuous scores. The effect size for linear regressions represents the beta coefficient with confidence interval. Abbreviations: ICU: intensive care unit, CI: confidence interval, IES-R: Impact of Event Scale-Revised, HADS: hospital anxiety and depression, EQ-5D: European Quality of life – 5 dimensions, VAS: visual analog scale.

### References

1. von Elm E, Altman DG, Egger M, Pocock SJ, Gotzsche PC, Vandenbroucke JP, Initiative S: **Strengthening the Reporting of Observational Studies in Epidemiology (STROBE) statement: guidelines for reporting observational studies**. *BMJ* 2007, **335**(7624):806-808.

2. Decavèle M, Similowski T, Demoule A: **Detection and management of dyspnea in mechanically ventilated patients**. *Current Opinion in Critical Care* 2019, **25**(1):86-94.

3. Demoule A, Decavele M, Antonelli M, Camporota L, Abroug F, Adler D, Azoulay E, Basoglu M, Campbell M, Grasselli G *et al*: **Dyspnoea in acutely ill mechanically ventilated adult patients: an ERS/ESICM statement**. *Intensive Care Med* 2024, **50**(2):159-180.

4. Demoule A, Hajage D, Messika J, Jaber S, Diallo H, Coutrot M, Kouatchet A, Azoulay E, Fartoukh M, Hraiech S *et al*: **Prevalence, Intensity and Clinical Impact of Dyspnea in Critically Ill Patients Receiving Invasive Ventilation**. *Am J Respir Crit Care Med* 2022, **205**(8):917-926.

5. Pham T, Heunks L, Bellani G, Madotto F, Aragao I, Beduneau G, Goligher EC, Grasselli G, Laake JH, Mancebo J *et al*: **Weaning from mechanical ventilation in intensive care units across 50 countries (WEAN SAFE): a multicentre, prospective, observational cohort study**. *Lancet Respir Med* 2023, **11**(5):465-476.

6. Bergeron N, Dubois MJ, Dumont M, Dial S, Skrobik Y: **Intensive Care Delirium Screening Checklist: evaluation of a new screening tool**. *Intensive Care Med* 2001, **27**(5):859-864.

7. Ely EW, Margolin R, Francis J, May L, Truman B, Dittus R, Speroff T, Gautam S, Bernard GR, Inouye SK: **Evaluation of delirium in critically ill patients: validation of the Confusion Assessment Method for the Intensive Care Unit (CAM-ICU)**. *Crit Care Med* 2001, **29**(7):1370-1379.

8. Sessler CN, Gosnell MS, Grap MJ, Brophy GM, O'Neal PV, Keane KA, Tesoro EP, Elswick RK: **The Richmond Agitation-Sedation Scale: validity and reliability in adult intensive care unit patients**. *Am J Respir Crit Care Med* 2002, **166**(10):1338-1344.

9. Weiss DS: **The impact of event scale-revised**. *International and cultural psychology* 2007:219-238.

10. de Miranda S, Pochard F, Chaize M, Megarbane B, Cuvelier A, Bele N, Gonzalez-Bermejo J, Aboab J, Lautrette A, Lemiale V *et al*: **Postintensive care unit psychological burden in patients with chronic obstructive pulmonary disease and informal caregivers: A multicenter study**. *Crit Care Med* 2011, **39**(1):112-118.

11. Zigmond AS, Snaith RP: **The hospital anxiety and depression scale**. *Acta Psychiatr Scand* 1983, **67**(6):361-370.

12. Jutte JE, Needham DM, Pfoh ER, Bienvenu OJ: **Psychometric evaluation of the Hospital Anxiety and Depression Scale 3 months after acute lung injury**. *J Crit Care* 2015, **30**(4):793-798.

13. M MV, K MV, S MAAE, de Wit GA, Prenger R, E AS: **Dutch Tariff for the Five-Level Version of EQ-5D**. *Value Health* 2016, **19**(4):343-352.

14. Herdman M, Gudex C, Lloyd A, Janssen M, Kind P, Parkin D, Bonsel G, Badia X: **Development and preliminary testing of the new five-level version of EQ-5D (EQ-5D-5L)**. *Qual Life Res* 2011, **20**(10):1727-1736.

15. EuroQol G: **EuroQol--a new facility for the measurement of health-related quality of life**. *Health Policy* 1990, **16**(3):199-208.

16. Andrinopoulou ER, Rizopoulos D, Jin R, Bogers AJ, Lesaffre E, Takkenberg JJ: **An introduction to mixed models and joint modeling: analysis of valve function over time**. *Ann Thorac Surg* 2012, **93**(6):1765-1772.

17. Watanabe S: **Asymptotic Equivalence of Bayes Cross Validation and Widely**

**Applicable Information Criterion in Singular Learning Theory**. *Journal of Machine Learning Research* 2010(11):3571-3594.

18. Rizopoulos D: **Github: Univariate and Multivariate Joint Models**. 2024.
